# The complete mitochondrial genome of *Coronocyclus labratus* (Rhabditida: Cyathostominae)

**DOI:** 10.1080/23802359.2020.1721361

**Published:** 2020-02-03

**Authors:** Shuwen Yang, Peng Li, Chenguang Sun, Wei Liu

**Affiliations:** a College of Life Sciences, Henan Normal University, Xinxiang, China;; b Key Laboratory of Environment Change and Resources Use in Beibu Gulf, Nanning Normal University, Nanning, China

**Keywords:** *Coronocyclus labratus*, mitochondrial genome, phylogenetic analysis

## Abstract

The complete mitochondrial genome sequence of *Coronocyclus labratus* was sequenced in the present study. It was determined to be 13,856 bp in length, containing 12 protein-coding genes, 2 ribosomal RNA genes, 22 transfer RNA genes, and 2 non-coding regions. The nucleotide sequence data of 12 protein-coding genes of *C. labratus* and other 16 Strongylidae species were used for phylogenetic analyses. *Coronocyclu labratus* formed a monophyletic cluster with the remaining Strongylidae species. *Coronocyclu*s genus was present in the same clade with high statistical support. The mitogenome sequences will facilitate taxonomy as well as systematic studies of Cyathostominae nematodes.

Cyathostominae and Strongylinae are the two subfamilies of Strongylidae nematodes, which inhabit in the large intestine with a high prevalence in *Equus* species. They cause series clinical symptoms such as anemia, weight loss, and even death (Lichtenfels et al. 2002). *Coronocyclus labratu*, which belongs to subfamily Cyathostominae, is a significant Strongylidae nematode. In the present study, we assembled and characterized the complete mitochondrial genome of *C. labratus* from horse and to reconstruct the phylogenetic relationships of Strongylidae nematodes.

Specimen (Specimen Voucher: Biological Specimen Museum of Henan Normal University #YJ0096) were isolated from intestine of infected donkey in Yanjin, Henan Province, China (114°19′E, 33°14′N). Genomic DNA of the specimen was extracted using standard phenol/chloroform methods (Sambrook et al. 1989). Illumina HiSeq 4000 platform were used to amplify the entire mitochondrial genome sequence (GenBank accession number: MN832736). The complete mitogenome sequence of *C. labratus* was assembled with SOAPdenovo v2.04 (Luo et al. 2012) and MITObim v1.6 (Christoph et al. 2013). The complete mitogenome was annotated using the DOGMA (Wyman et al. 2004) and tRNAscan-SE (Lowe and Eddy 1997).

The complete mitochondrial genome of *C. labratus* is 13,856 bp in length. The complete mitochondrial genome has 12 protein-coding genes, 22 transfer RNA genes (tRNA), and 2 ribosomal RNA (rRNA, rrnL, and rrnS) genes, all genes are encoded by the same strand. Two kinds of start codons (ATT and TTG) and two kinds of termination codons (TAA and T) were used in the 12 PCGs, respectively. The studied genome has a high T content (45.62%) and a low C content (6.96%), resulting in a very strong A + T bias (76.91%) and in particular, the AT-rich region (85.63%). The size of the 22 tRNAs ranging from 52 to 63 bp. The rrnL (975 bp) is located between trnH and nad3, whereas the rrnS (701 bp) is located between trnE and trnS(UCN).

Phylogenetic analyses were conducted using the Bayesian inference (BI), implemented in MrBayes version 3.1.2 (Ronquist and Huelsenbeck 2003). Mitogenome sequences of 16 other Strongylidae species were retrieved from GenBank. Analyses were performed using 12 protein-coding genes, using *Bunostomum phlebotomum and Ancylostoma tubaeforme* as outgroup. Phylogenetic analysis showed that *Coronocyclu*s *labratus* formed a monophyletic cluster with the remaining Strongylidae species. *Coronocyclu*s genus was present in the same clade with high statistical support (Figure 1). Strongylidae nematodes grouped into two major clades: *Strongylus* species and others. Species of genus *Triodontophorus* (*T. brevicauda, T. serratus, and T. nipponicus*) clustered together with species in subfamily Cyathostominae, although they belonged to subfamily Strongylinae. The dendrogram topology is highly congruent with previous studies (Gao. et al. 2017; Li et al. 2019). The mitogenome sequences will facilitate taxonomy as well as systematic studies of Cyathostominae nematodes in the future.

**Figure 1.**
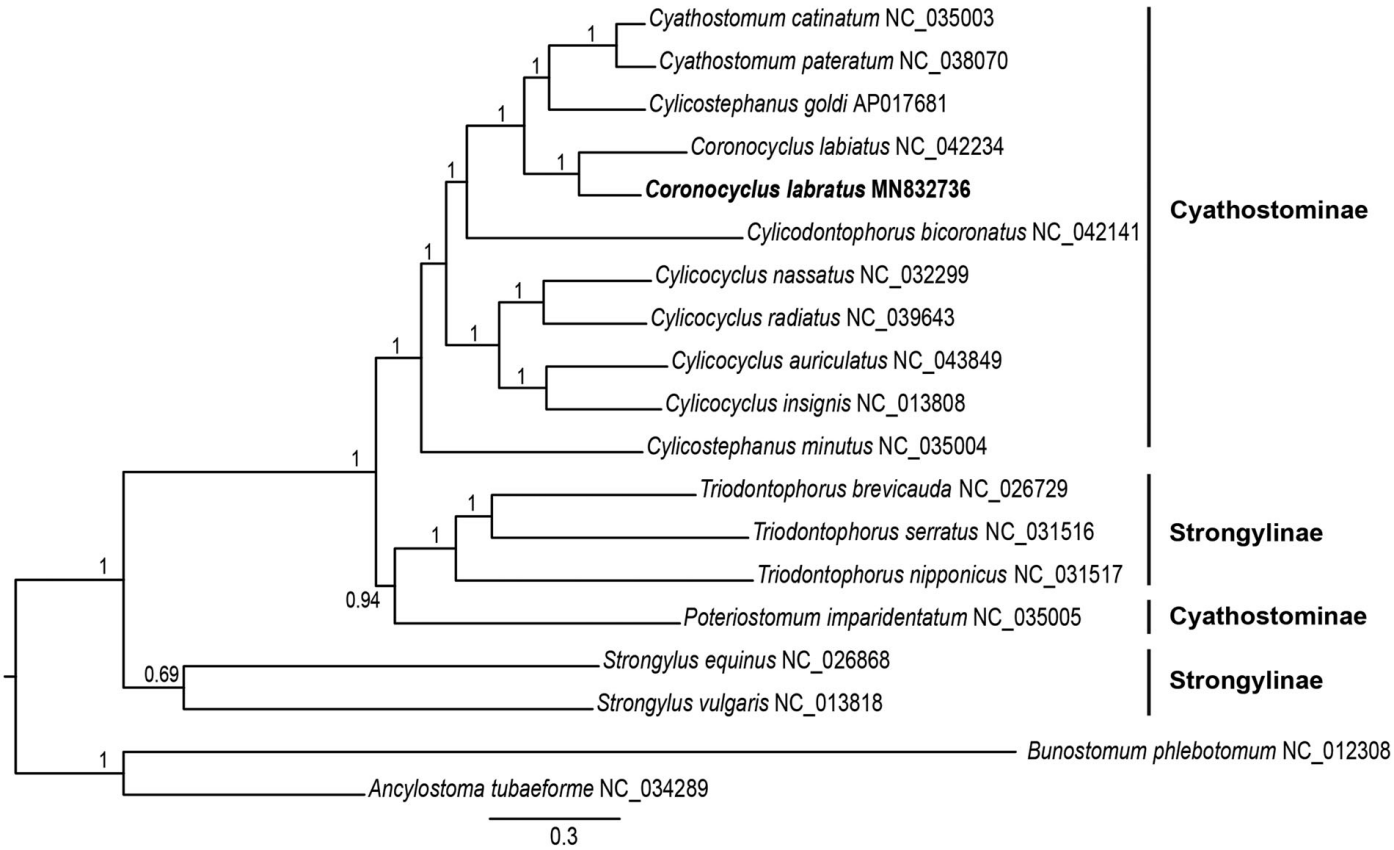
Phylogenetic relationships of *Coronocyclus labratus* and other 16 Strongylidae nematodes based on 12 protein-coding genes were analyzed with Bayesian inference (BI) method. *Bunostomum phlebotomum* and *Ancylostoma tubaeforme* were used as outgroups.
